# COCONUT 2.0: a comprehensive overhaul and curation of the collection of open natural products database

**DOI:** 10.1093/nar/gkae1063

**Published:** 2024-11-26

**Authors:** Venkata Chandrasekhar, Kohulan Rajan, Sri Ram Sagar Kanakam, Nisha Sharma, Viktor Weißenborn, Jonas Schaub, Christoph Steinbeck

**Affiliations:** Institute for Inorganic and Analytical Chemistry, Friedrich Schiller University Jena, Lessingstr 8, 07743, Jena, Germany; Institute for Inorganic and Analytical Chemistry, Friedrich Schiller University Jena, Lessingstr 8, 07743, Jena, Germany; Institute for Inorganic and Analytical Chemistry, Friedrich Schiller University Jena, Lessingstr 8, 07743, Jena, Germany; Institute for Inorganic and Analytical Chemistry, Friedrich Schiller University Jena, Lessingstr 8, 07743, Jena, Germany; Institute for Inorganic and Analytical Chemistry, Friedrich Schiller University Jena, Lessingstr 8, 07743, Jena, Germany; Institute for Inorganic and Analytical Chemistry, Friedrich Schiller University Jena, Lessingstr 8, 07743, Jena, Germany; Institute for Inorganic and Analytical Chemistry, Friedrich Schiller University Jena, Lessingstr 8, 07743, Jena, Germany

## Abstract

The COCONUT (COlleCtion of Open Natural prodUcTs) database was launched in 2021 as an aggregation of openly available natural product datasets and has been one of the biggest open natural product databases since. Apart from the chemical structures of natural products, COCONUT contains information about names and synonyms, species and organism parts in which the natural product has been found, geographic information about where the respective sample has been collected and literature references, where available. COCONUT is openly accessible at https://coconut.naturalproducts.net. Users can search textual information and perform structure, substructure, and similarity searches. The data in COCONUT are available for bulk download as SDF, CSV and a database dump. The web application for accessing the data is open-source. Here, we describe COCONUT 2.0, for which the web application has been completely rewritten, and the data have been newly assembled and extensively curated. New features include data submissions by users and community curation facilitated in various ways.

## Graphical Abstract

**Graphical Abstract:**
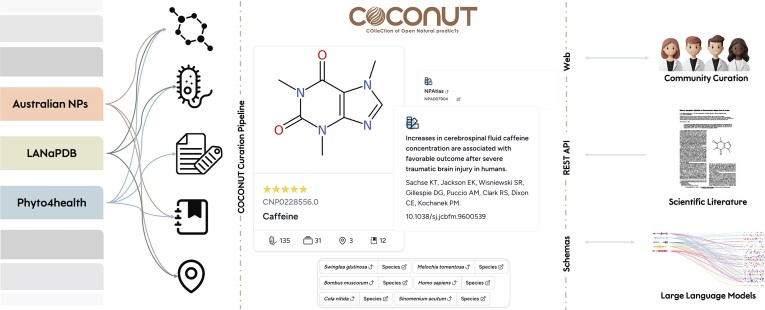

## Introduction

Natural products have long been recognized as a rich source of biologically active compounds. They form the basis of many therapeutic agents and play a role in drug discovery and development (1). Natural products’ structural diversity and complexity often result in unique biological activities, making them interesting starting points in the search for novel medicines. However, the sheer volume of natural products identified and characterized in various publication formats – from scientific literature to patents – presents a significant challenge regarding data organization, accessibility and utility. As a result, a comprehensive, well-curated and open database of natural products is required for researchers wishing to exploit the potential of these compounds in scientific and industrial applications (2).

The COCONUT (COlleCtion of Open Natural prodUcTs) database has been developed to address this need by providing an extensive, freely accessible repository of natural product data (3). Recognizing the growing importance of open-access resources in the scientific community, COCONUT compiles data from various sources, providing detailed information on hundreds of thousands of natural products. However, as the volume and complexity of natural product data continue to grow, there is an urgent need to enhance the database’s capabilities to keep pace with the evolving needs of the scientific community.

In response to this challenge, we have undertaken a complete re-engineering of the COCONUT database. This significant update includes a major overhaul of the underlying software architecture and the incorporation of new source collections of natural product data. Our efforts have focused on improving the database’s scalability, reliability and usability, ensuring that it can accommodate the growing dataset while providing robust performance for users. Being a product of accumulation from various source databases, the initial compilation contained and in part still contains incorrect information from the source databases and in particular non-natural products. We removed obvious cases such as fluorinated compounds immediately, but still invalid entries will remain. A key feature of the updated COCONUT database is therefore the newly implemented curation interface. This interface allows for more efficient and accurate data curation, in particular by users, enabling continuous refinement of database entries and ensuring that users can access the most accurate and up-to-date information. The curation process facilitates the integration of user feedback thus allowing the community to contribute to the ongoing improvement of the database.

In summary, the renovated COCONUT database is a more comprehensive, curated and user-friendly resource. We aim to enable researchers to explore the vast chemical space of natural products more effectively. This will ultimately accelerate the discovery of new bioactive compounds and contribute to advancing science and medicine. COCONUT 2.0 is accessible at https://coconut.naturalproducts.net.

## Database features and functionality

The 2019 version of COCONUT aggregates 53 openly accessible, often specifically focused natural product databases into a general information source. With the updated COCONUT version reported here, we move further than this initially envisioned purpose. COCONUT database 2024 serves as a platform that advances natural product research, providing data and tools to deposit, curate and reuse natural product data while adhering to FAIR (Findable, Accessible, Interoperable and Reusable) principles (4) for research data management. Additional emphasis has been placed on provenance data and semi-automated curation to ensure high data quality, which is essential in data-driven research. The new community curation feature in COCONUT empowers researchers worldwide to contribute and refine data, enhancing the database’s accuracy, richness and relevance, making it a powerful collaborative resource for natural product research. To ensure the accuracy and integrity of community curation, COCONUT now includes a detailed audit log of all actions and updates, ensuring traceability and accountability.

### Data

**Figure 1.**
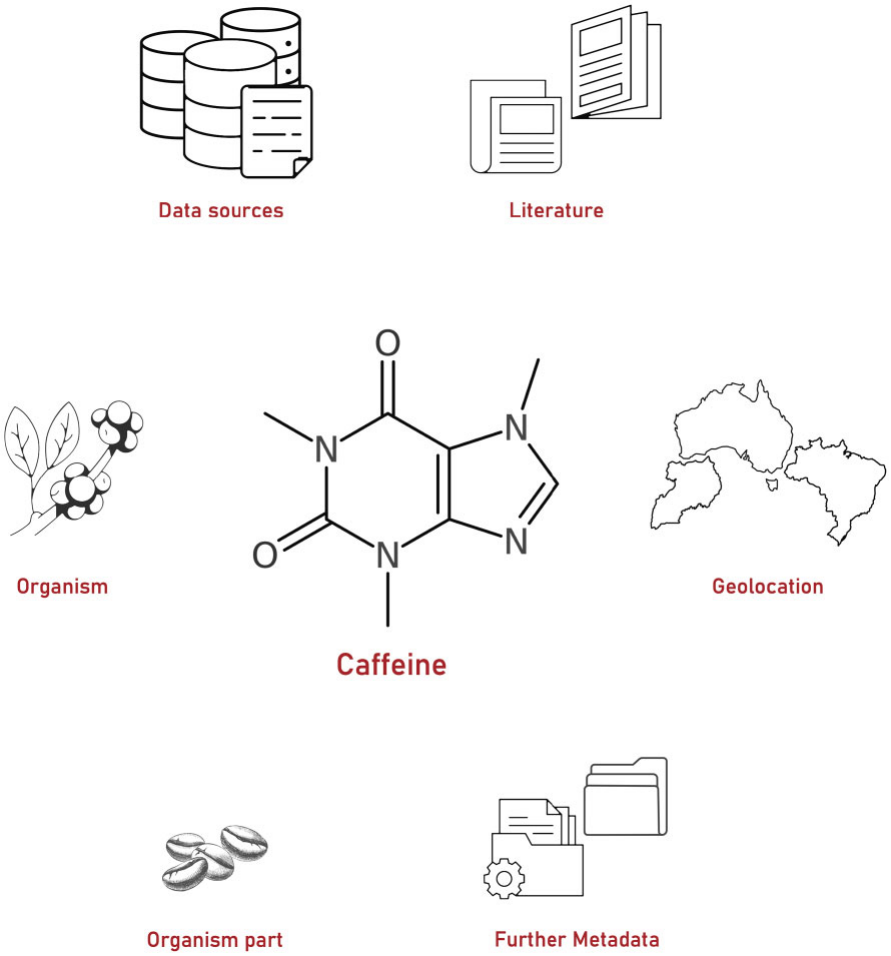
Components of an exemplary database record in COCONUT 2.0, which includes its source organism, organism part, chemical structure, geographic information, associated literature, data source collections and further metadata.

COCONUT continues to serve as a source of information about natural products’ chemical structures as shown in Figure 1. We focus on a core set of structural properties and annotations with external links to resources containing additional information. This core set comprises the chemical structure of the natural product, names and synonyms, species and organism part in which it has been found, geographic information about where the respective sample has been collected and literature references supporting the given information. Further data that can be computed from the compound’s structure are also provided. Examples are a natural product likeness score (5), the presence or absence of sugar moieties (6) or Lipinski’s rule of five violations (7).

### Collections

COCONUT 2.0 focuses on displaying and allowing users to search for molecules within the database, particularly identifying their origin in specific datasets. As some molecules are unique to individual databases, it is advantageous to trace their provenance and understand how they were included in particular datasets. To support this, COCONUT aggregates molecules and can present them under the collection name from which they were originally retrieved. Additionally, each collection will be assigned a citable DOI, enabling researchers to submit their original datasets to COCONUT for inclusion and display. This way, individual natural product research groups, for example, can start and maintain a personal collection in COCONUT, showcasing structures they have elucidated and published. Initiatives dedicated to collecting natural product information from specific geographic locations or from particular areas of the Tree of Life can maintain their dedicated collections. COCONUT 2.0 already includes such collections.

### Search

Scientists typically use COCONUT to look up individual structures by name or by structure searches. COCONUT’s advanced search page allows users to draw a chemical structure in a structure editor (8) and perform a dereplication through an exact match search. One can also perform a substructure or a similarity search. Apart from structure names or structure representations like SMILES, other textual data can be searched in COCONUT. For example, all compounds reported for a given organism or assigned a specific chemical class can be retrieved. Furthermore, all natural products linked to a specific scientific article or other literature references can be queried.

### Compound card

**Figure 2.**
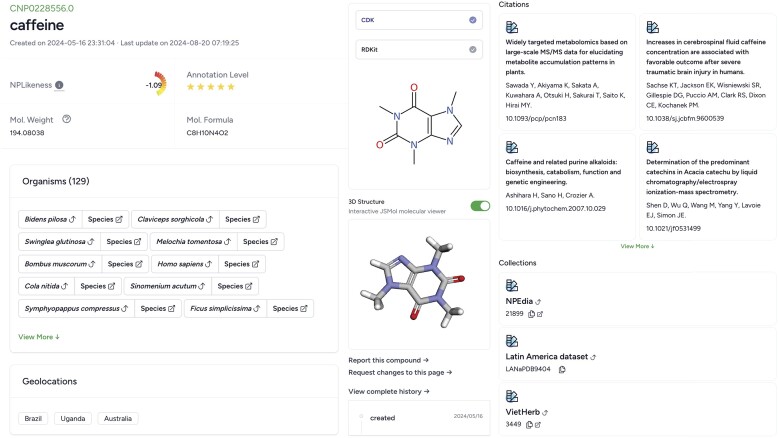
A compound card entry for caffeine as presented in COCONUT 2.0, showcasing its layout. The compound view includes the NPLikeness score, annotation level, molecular properties, 2D structure and an interactive 3D molecular viewer. Additional details highlight species associations, geolocations and literature references. Furthermore, the card provides links to collections that trace back to the original source datasets, ensuring data provenance, along with an audit trail documenting the entry’s history.

The compound page details the core structural properties and annotations (organism, sample location, geolocation, citations and data sources with associated identifiers) organized in sections (see Figure 2). External and internal links are provided when available to facilitate seamless browsing. For example, organism information on the compound details page offers internal links to retrieve all natural products associated with the organism on the platform as well as external links mapping the organism to corresponding ontology terms representing species taxonomies. Molecular 2D and 3D representations are rendered on the page for visual inspection of the molecule structure. Cahn-Ingold-Prelog (9) annotations are labelled (chiral centres are labelled R or S for defined stereochemistry and with a question mark (?) for unspecified stereochemistry) by default in the 2D representation to specify the stereo configuration of the chiral molecule uniquely. Information on computed molecular descriptors and chemical classification generated by ClassyFire (10) is also provided when available. The compound details page also presents options for users to report structures they deem to be of synthetic and not natural origin or request changes to the existing data on the website (see below).

### Community submission and curation

A novel feature in COCONUT 2.0 is largely automated data submission and curation. Users can submit structures and metadata to COCONUT that have been newly reported in the scientific literature or have not yet been included in the database. We provide a CSV format template for bulk submission of multiple data additions or updates. These will be automatically parsed, standardized and included in COCONUT after approval by the curators. Data curation can also be performed through tickets created from the pages of individual compounds, where users can flag compounds needing curation or suggest concrete changes to a compound’s entry. A specific use case we envision is that of users reporting structures in the database that they would not classify as natural products but rather as synthetic compounds. Since most studied organisms in the Anthropocene come into contact with synthetic chemicals almost everywhere every time, these regularly appear in natural product databases as well. This reporting feature requires users to provide evidence to support their claims. Submitted reports are internally validated by curators on the platform (internal and community), and the data are updated accordingly. Updates are audited, and the complete history of the data changes (who, what and when) is accessible on the compound details page. Furthermore, a link to COCONUT’s GitHub issue tracker is prominently displayed at the border of every COCONUT page, allowing users to submit reports on bugs or make feature requests.

### Downloads and programmatic data access

The entire COCONUT content can also be downloaded in various formats, including a CSV file, an SD File or a full database SQL dump. This enables researchers to perform local and offline bulk data analyses and access all compound metadata fields. The SQL dump contains every metadata field since it is a direct representation of the COCONUT data model. The SDF and CSV exports are available in two flavours, one that contains only the structural data and one that provides most annotation data in addition to the structural data. The downloads page also contains a use-case section providing further CSV files for various applications.

To assist mass spectrometry data analyses for metabolomics, a CSV file containing the natural product structures with their molecular formulae and mono-isotopic masses is presented. Additionally, species and taxonomy annotations in COCONUT can be used to compile specific suspect lists for metabolite identification workflows. Another CSV file download option includes pre-computed molecular descriptor values for each compound, like synthetic feasibility (11) and NPLikeness score (5), to prioritize favourable structures in virtual screening campaigns. Further examples are CSV files containing specific substructures generated from the entire natural product collection. These contain all functional groups identified in the COCONUT natural product structures according to the Ertl algorithm for functional group identification (12,13) and how often they appear in the dataset, the same for the main molecular scaffolds of the database structures, and a third file with scaffolds and smaller parent scaffolds generated according to the scaffold tree algorithms (14,15). They were generated using the open MORTAR (MOlecule fRagmenTAtion fRamework) application for *in silico* molecule fragmentation (16) and can help in approaches like fragment-based drug discovery and to gain a general overview of the most prominent substructures in natural products.

COCONUT 2.0 infrastructure facilitates real-time updates for new natural product submissions, reported existing compounds and requested and approved data changes. Monthly data releases are accessible on the COCONUT downloads page to track these modifications over time. Those monthly releases are also deposited on Zenodo with release notes containing a change log. In addition, the REST API (see below) provides real-time data, guaranteeing users constant access to the most up-to-date information.

#### REST API

The COCONUT database also provides a REST API through which users can programmatically access and interact with data and track changes with each successive data release. This API offers endpoints for querying chemical structures, retrieving associated metadata and audit information and accessing computed properties. Compliant with OpenAPI specifications (17), the COCONUT REST API ensures standardized interactions, thereby enhancing accessibility and interoperability with various tools and platforms. The API documentation is accessible at https://coconut.naturalproducts.net/api-documentation.

#### Semantic markup

COCONUT integrates Bioschemas (18,19) markup into its web resources to enhance data interoperability and discoverability. By adhering to these standards, COCONUT improves the findability of natural product data, facilitating more efficient indexing by search engines and other services. An example scheme of a COCONUT record can be found at https://coconut.naturalproducts.net/api/schemas/bioschemas/CNP0606256.0.

### Documentation

The COCONUT database provides documentation to help users navigate, search, download, report and request data updates on the platform. The documentation covers instructions for submitting and curating data through the curation interface. The curation pipeline and database schema details are meticulously documented to ensure transparency and community feedback on the adapted procedures.

Dedicated sections provide step-by-step instructions for developers interested in contributing to the platform and those who want to set up a local instance. These resources are regularly updated to incorporate the latest developments and user feedback, making them indispensable for new and experienced users. The COCONUT web interface documentation can be found at https://steinbeck-lab.github.io/coconut/introduction.html.

## Data additions and curation

The COCONUT 2021 release aggregated data extracted from 53 data sources and several manually curated entries collected from the literature, all re-imported into COCONUT 2.0 using the new curation pipeline and database model. Some of these data sources have released new versions or metadata updates between 2021 and 2024. These include sources such as BIOFACQUIM (20), InflamNat (21), Carotenoids Database (22), FooDB (23), KnapSaCK (24), NANPDB (25), NPedia (26), NPAtlas (27), NPASS (28), PubChem NPs (29), Exposome-explorer (30), Spektraris NMR (31), InPACdb (32), SANCDB (33), Seaweed Metabolite Database (34), StreptomeDB (35), Supernatural 3 (36), TIPdb (37) and TPPT (38). They are processed and included in COCONUT 2.0 in addition to the previous releases. For collections that are offline and no longer accessible (13 sources), data from the COCONUT 2021 release is re-imported. Data sources not updated since 2021 have also been imported from the COCONUT 2021 release. COCONUT 2.0 now comprises an extensive collection of natural products from 63 data collections (see Supplementary 1).

The newly imported data sources include geographically specific datasets such as the Australian Natural Products dataset (39), the Latin American Natural Product Database (40), the Phyto4Health Database of Phytocomponents from Russian Pharmacopoeia Plants (41) and the African Natural Products Database (42). There are also sources dedicated to a particular source organism, such as Watermelon DB for *Citrullus lanatus* (43). Other sources included in the latest version of COCONUT are CMNPD (44), EMNPD (45) and CyanoMetNP (46).

Data from the sources were downloaded in bulk whenever the sources offered a bulk download option. During the extraction process, it was observed that these bulk downloads often did not include additional metadata, such as organism details and citation information. To address this, web scrapping was used to mine individual molecular entry pages and gather the necessary information. It was also employed to extract the data from data sources that did not provide a bulk download and only displayed entries as HTML pages. All the gathered information was then harmonized and saved. Structural data from these sources are initially parsed using RDKit (47), and molecules that fail to be parsed are discarded. Successfully parsed entries are then saved as a CSV file with associated metadata, such as name, CAS ID (48), organism, sample location, synonyms, citation and geolocation when available. The CSV files are then loaded into COCONUT as separate collections to preserve provenance data. Subsequently, the molecules are processed by the ChEMBL Structure Curation Pipeline *Checker* (49) to verify the validity of the chemical structures and identify any serious problems like valence errors. Molecules flagged with *Checker* error codes six or higher are marked as failed entries and filtered at this stage (if necessary, these failed entries can be manually reviewed, corrected and resubmitted). Those that successfully pass the *Checker* are advanced to the next processing step.

Approximately 1.5 million structure entries are imported into COCONUT across all data sources. After removing failed entries, the molecular structures are standardized with the ChEMBL pipeline *Standardizer* module. Then, synthetic compounds such as fluorine-containing compounds, sulfonamides [We are aware of the rare exceptions for these compound classes. Natural products, which have been reported to contain these functional groups, will be added in the future] or drug derivatives, and entries containing multiple organic structures (multi-component entries) are removed, and a total of 1022 536 molecules are imported into the COCONUT table structure. The canonical isomeric kekulized SMILES representation (50,51) is generated for each molecule and used to determine its uniqueness and combine duplicates.

Since different stereoisomers of a molecule can have distinct biological activities, they are treated as different molecules in the COCONUT database. Many structures imported in the above step had stereocenters, but those were left undefined by the sources. Structures with completely undefined stereocenters that had well-defined stereo variants from other sources were removed, leading to 695 133 unique natural product structures in the September 2024 release of the COCONUT database. They include 82 220 molecules without stereocenters, 539 350 molecules with preserved stereochemistry and 73 563 molecules with stereocenters but with absolute stereochemistry not defined.

COCONUT identifiers (‘CNP’ prefix and 7 digits) issued previously are mapped and preserved to resolve to the same molecular entity from COCONUT 1.0, and new identifiers are issued to new entries. Stereochemical variants of the same molecule are grouped under the same identifier and are issued a unique postfix to the COCONUT identifier.

The COCONUT curation pipeline automatically processes and maps metadata from data sources. For new entries, the natural product name is imported from the data source. For molecules already existing in the database, the name from the data source is added to the list of synonyms. Organism and sample location information, when available, are mapped to ontology terms using the EMBL-EBI Ontology Lookup Service (OLS4) (52) or the Global Names Finder API (IRI and rank) (53). Citation information and provenance data are imported and mapped based on DOI. Europe PMC Restful Web Service (53,54) and Crossref REST API (55) are used to fetch citation metadata. Geolocation, synonyms, CAS ID, IUPAC names and other metadata are imported and mapped. Using Chemistry Development Kit (CDK) (56,57) and RDKit, a range of molecular properties, descriptors and fingerprints are computed. Each molecular entry was given an NPLikeness score (5) implemented in RDKit, a metric used to assess how closely a given compound resembles a natural product.

The chemical classification of all natural products in COCONUT is performed with ClassyFire (10) and, when successfully generated, is displayed in the corresponding section of the compound details page.

In COCONUT, each natural product is assigned a score (annotation score, see Supplementary 2) to indicate its level of annotation. The scoring algorithm evaluates a molecule by assigning scores based on the presence and quantity of key attributes, such as literature references, taxonomic provenance annotation, CAS number, synonyms, names and data sources. It then calculates a weighted total score, scales it and rounds up the final score to the nearest integer for standardized assessment (1 star is the lowest quality annotation, while 5 stars is the highest quality). A histogram of the annotation score value distribution in COCONUT 2.0 is given in supplementary material (Supplementary 2).

**Figure 3.**
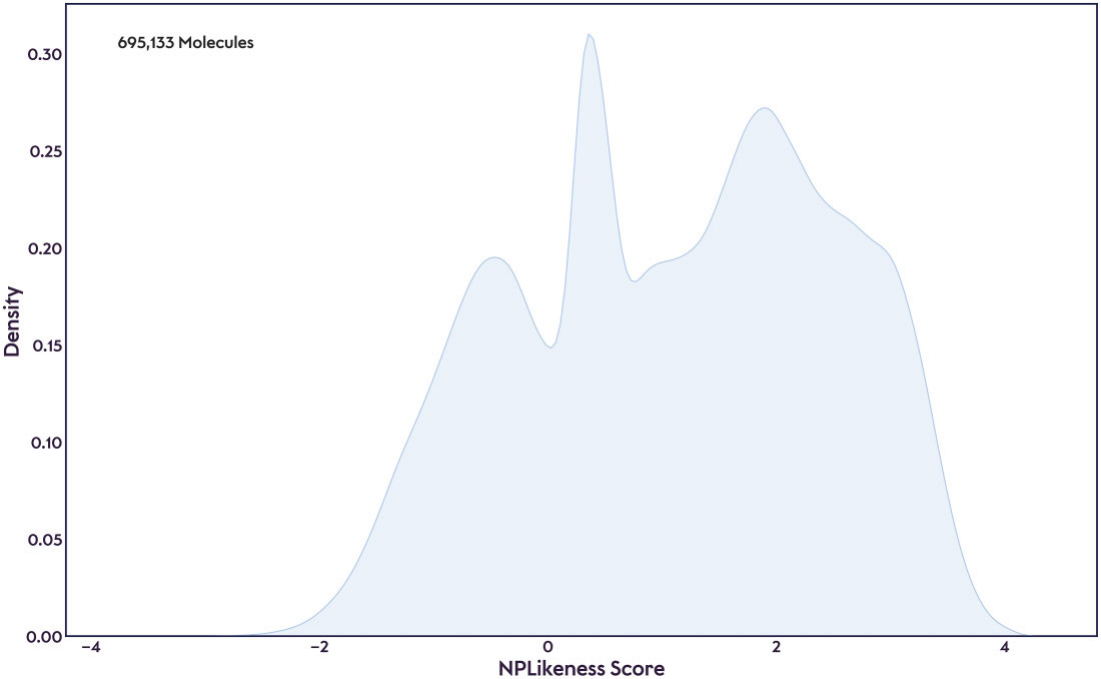
NPLikeness score distribution in COCONUT 2.0 (September 2024).

Combining and curating data from multiple open natural product collections for COCONUT 2.0 has highlighted several issues with circular imports between databases. Many of these aggregate data from other collections and are often, in turn, aggregated themselves by other collections as well. This way, misclassified structures, like synthetic compounds or drug derivatives, or compounds with structural problems, such as valence errors, propagate across datasets and database versions. The incorporation of an audit trail feature and well-annotated provenance information in COCONUT enables users to identify and effectively resolve these problems through community curation. To assess the current curation state of COCONUT 2.0 (September 2024), the NPLikeness score distribution for all compounds listed in the dataset is given in Figure 3. The distribution is characterized by a multimodal pattern, with prominent peaks observed in the NPLikeness score ranges of −2 to 0, 0 to 1 and around 2. This suggests the coexistence of both natural products and non-natural products, or rather synthetic compounds, within the dataset. The substantial density below zero and the complex distribution pattern underscore the need for a comprehensive curation process to refine the database and remove non-natural products, address potential misclassifications and ultimately improve COCONUT’s reliability as a comprehensive natural product resource. Examples of synthetic compounds that were previously listed in COCONUT but are now revoked are given in Supplementary 3. It should be noted though that a substantial number of natural products with a negative NPLikeness score is known – caffeine with an NPLikeness score of −1.09 is a prominent example.

## Architecture

The COCONUT 2.0 platform is designed based on a microservices architecture, leveraging pre-existing open-source components as much as possible to make it secure, scalable and sustainable. Each microservice is encapsulated in Docker containers to ensure independence from the host environment. Microservices allow for future exchange of software components without the need for extensive code modifications.

### Deployment

COCONUT can be deployed across multiple nodes using Kubernetes (58). Kubernetes (K8s) is an open-source container orchestration software for managing computing clusters. K8s enables the efficient sharing of computing resources across multiple processes, optimizing infrastructure utilization by dynamically adjusting resource allocation based on demand. COCONUT Helm charts are developed and distributed for easy installation and upgrades.

While K8s can manage containers deployed across multiple nodes, COCONUT can also be run on a single host system using Docker Compose if required (part of the standard distribution).

COCONUT’s software development and deployment lifecycle (code analysis and linting, running tests and builds in isolated environments, generating documentation and publishing packages) is streamlined with Continuous Integration and Continuous Delivery workflows integrated with GitHub.

### Database

The COCONUT data structure is clearly defined and has many-to-many or one-to-many relationships across the data models, such as molecules, organisms, citations and collections. To gain greater flexibility and control over data integrity, we migrated from MongoDB to PostgreSQL, a relational database. It also leverages the RDKit PostgreSQL cartridge to enable similarity and substructure searches. The COCONUT database schema is available online at https://www.figma.com/board/yVQeNRsqlkXOgI5BIlMlb4/COCONUT_DB?node-id=0-1&node-type=CANVAS.

### Cache

A significant portion of COCONUT data remains static until new annotations or data updates occur. Caching is employed to avoid repeating the same database queries and improve performance. COCONUT employs Redis-based caching (59) to store and serve data from memory. Data are cleared from the cache and updated when the underlying data models update.

### Job queues

To avoid heavy usage impacting the web interface performance, new compound submissions to COCONUT are handled by background jobs managed by Redis job queues. These are executed on scalable worker pods. This job batching feature allows the COCONUT compound submission and curation system to handle hundreds of molecules per submission. Because of the microservice architecture, the underlying technology behind caching and queues can be easily swapped with alternative solutions based on resource availability.

### Frontend

The COCONUT frontend user interface is developed using Livewire (60), a PHP framework for writing highly interactive web apps, and AlpineJS (61). This lightweight JavaScript library makes adding client-side interactivity to web pages easy.

These frameworks are chosen primarily to enable search engine optimization and lazy loading. This way, the COCONUT frontend stack does not require server-side rendering workarounds to enable access to search engine crawlers. Tailwind CSS (62), an open-source utility-first CSS framework, is used to design COCONUT interfaces and ensure responsiveness across different screen sizes. The chemical structure editor for the search functions is powered by the OpenChemLib (8) library.

### Backend

The COCONUT backend comprises multiple microservices that perform specific tasks and communicate with each other to respond to web, command line and API requests. COCONUT’s web application is developed using Laravel (63). This PHP web application framework provides a range of features, including jobs, queues, scheduled tasks and notifications, as a part of its standard functionality. Cheminformatics Microservice (64) is an in-house developed microservice that provides a unified interface to access commonly used functionalities from various cheminformatics toolkits, including RDKit, CDK and Open Babel (65). It is deployed alongside the web application and scaled on-demand to serve requests such as 2D and 3D molecule rendering from the web application and molecular representations standardization for submission and curation pipelines. All the COCONUT curation routines are integrated within the Cheminformatics Microservice.

## Feature comparison between COCONUT version 1.0 and 2.0

COCONUT 2.0 aims to become a comprehensive and reliable resource of high-quality natural product data. Unlike version 1.0, it now features extensive options for its user community to report issues with data entries. Registered curators can alter data directly. All changes are recorded in a change history. This updated version focuses on offering standardised metadata, encompassing detailed information on organisms, their geographic location, origins and references to sources and literature. The metadata is aligned with relevant ontology terms, improving the dataset’s accessibility and usability for researchers. Structural data from the diverse collections in COCONUT 2.0 is standardised using the ChEMBL curation pipeline and RDKit to maintain data consistency. As of September 2024, the number of data sources has increased from 53 to 63, by incorporating new geographical and organism-specific datasets. The database has grown significantly, with the number of unique natural product structures reaching 695 133 in version 2.0. It will expand further as a result of the new community-driven data curation and submission features in COCONUT version 2.0. A more rigorous curation process is also employed to remove synthetic compounds, multi-component entries, valency errors, duplicate entries and fluorinated compounds. Table 1 lists the key feature improvements as an overview.

**Table 1.** Key feature improvements between COCONUT version 1.0 and 2.0

Feature	COCONUT 1.0	COCONUT 2.0 (September 2024)
Number of data sources	53	63
Data standardization		
Curation pipeline	ChEMBL curation pipeline with	ChEMBL curation pipeline with
	post-processing (CDK)	post-processing (RDKit)
Data models	Parent structure (without	Stereochemistry-aware (configuration
	stereochemistry)	preserved from the sources)
Source collection references	Not linked	Linked to online source collections
Organism details	Not mapped and only available in	Mapped to ontologies and taxonomic
	exports	classes. Available on the website and
		in exports.
Citations details	Not standardized or mapped	Mapped to DOIs, IBNs
Geographic information	Only available in exports	Available on the website and in
		exports
Curation features		
Community curation	Not available	Available
Reporting	Not available	Available
Audit trail	Not available	Available

## Conclusion and Outlook

The COCONUT natural products database has received an extensive update of its software infrastructure and data. The web interface has been completely redesigned to fulfil the community’s requirements and make it more resourceful in the ongoing advancement of natural product research. With the new version 2.0, the foundations are laid to move past the original idea to simply aggregate various open sources of natural product data in COCONUT towards establishing it as a resource that will be continuously improved and enriched by the community. Since COCONUT 2.0 has been accumulated from many source databases, it will inevitably contain false information. To support the creation of data of ever higher quality, COCONUT 2.0 includes new features like user reporting functionalities, data update requests and automatic data submission workflows.

Ongoing efforts focus on developing a workflow to enrich existing data and automatically curate novel and previously unreported natural products from newly published scientific literature. We envision developing an online literature monitoring system that can identify new publications reporting new natural product structures or new findings on known compounds using a specialized classifier. Such publications will be processed using a semi-automated literature extraction workflow. Users will also have the ability to upload natural product-related publications, which will be automatically scanned to check for any unrecorded structures. Such a system could employ fine-tuned Large Language Models (66) to accurately extract natural product structures and related metadata from a diverse range of relevant literature. These continuous efforts will greatly improve the database’s quality and its capacity to keep pace with the rapidly evolving field of natural product research.

## Supplementary Material

gkae1063_Supplemental_File

## Data Availability

The data underlying this article are available online at https://coconut.naturalproducts.net/download and on Zenodo at https://doi.org/10.5281/zenodo.13692394. The COCONUT database is accessible online at https://coconut.naturalproducts.net, and the latest version of the source code can be found in the GitHub repository at https://github.com/Steinbeck-Lab/coconut, also the archived version is available on Zenodo at https://zenodo.org/doi/10.5281/zenodo.13283948. The documentation is available at https://steinbeck-lab.github.io/coconut/.
