# Supplementary material for: COCONUT 2.0: a comprehensive overhaul and curation of the collection of open natural products database
Source: Nucleic Acids Res. 2024 Nov 26;53(D1):D634–43. doi: 10.1093/nar/gkae1063 (PMC11701633; doi:10.1093/nar/gkae1063)
Supplement: gkae1063_Supplemental_File [file gkae1063_supplemental_file.pdf]

Supplementary Data

Supplementary 1: Table of source collections aggregated in COCONUT 2.0:

Database name	Status as of 2024/09/05	Reference
AfroCancer	offline	https://doi.org/10.1021/ci5003697
AfroDB	offline	https://doi.org/10.1371%2Fjournal.pone.0078085
AfroMalariaDB	offline	https://doi.org/10.1186/s13588-014-0006-x
AnalytiCon Discovery NPs	online	https://ac-discovery.com/screening-libraries/
ANPDB	online	https://doi.org/10.1002/minf.202000163
Australian natural products	online	https://doi.org/10.25919/dh87-b149
BIOFACQUIM	online	https://doi.org/10.3390/biom9010031
BitterDB	online	https://doi.org/10.1093/nar/gky974
Carotenoids Database	online	https://doi.org/10.1093/database/bax004
ChEBI NPs	online	https://doi.org/10.1093/nar/gks1146
ChEMBL NPs	online	https://doi.org/10.1093/nar/gkw1074
ChemSpider NPs	online	https://doi.org/10.1021/ed100697w
CMAUP (Collective molecular activities of useful plants)	online	https://doi.org/10.1093/nar/gky965
CMNPD	online	https://doi.org/10.1093/nar/gkaa763
ConMedNP	offline	https://doi.org/10.1039/c3ra43754j
CyanoMetNP	online	https://doi.org/10.1016/j.watres.2021.117017
DrugBankNP	online	https://doi.org/10.1093/nar/gkx1037
EMNPD	online	https://doi.org/10.1186/s13321-023-00779-9
ETM (Ethiopian Traditional Medicine) DB	online	https://doi.org/10.1186/s12906-019-2634-1

**Exposome-explorer**	online	https://doi.org/10.1093/nar/gkw980
**FooDB**	online	https://doi.org/10.1007/978-1-4614-1689-0_11
**GNPS (Global Natural Products Social Molecular Networking)**	offline	https://doi.org/10.1038/nbt.3597
**HIM (Herbal Ingredients in-vivo Metabolism database)**	offline	https://doi.org/10.1186/1758-2946-5-28
**HIT (Herbal Ingredients Targets)**	offline	https://doi.org/10.1093/nar/gkq1165
**Indofine Chemical Company**	online	https://indofinechemical.com/Media/sdf/sdf_files.aspx
**InflamNat**	online	https://doi.org/10.1021/acs.jcim.8b00560
**InPACdb**	online	https://www.bioinformation.net/004/001500042009.htm
**InterBioScreen Ltd**	online	https://www.ibscreen.com/natural-compounds
**KNApSaCK**	online	https://doi.org/10.1093/pcp/pcs186
**Latin American Natural Product Database: LANaPDB**	online	https://doi.org/10.3390/ph16101388
**Lichen Database**	online	https://doi.org/10.1038/s41597-019-0305-1
**Marine Natural Products**	offline	https://doi.org/10.3390/md18040225
**Mitishamba database**	offline	http://erepository.uonbi.ac.ke/handle/11295/92273
**NANPDB (Natural Products from Northern African Sources)**	online	https://doi.org/10.1021/acs.jnatprod.7b00283
**NCI DTP data**	online	https://wiki.nci.nih.gov/display/NCIDTPdata/Compound+Sets
**NPACT**	online	https://doi.org/10.1093/nar/gks1047
**NPASS**	online	https://doi.org/10.1093/nar/gkac1069
**NPAtlas**	online	https://doi.org/10.1021/acscentsci.9b00806
**NPCARE**	offline	https://doi.org/10.1186/s13321-016-0188-5
**NPEdia**	online	https://doi.org/10.2751/jcac.7.157
**NuBBEDB**	online	https://doi.org/10.1038/s41598-017-07451-x
**p-ANAPL**	online	https://doi.org/10.1371/journal.pone.0090655
**Phenol-explorer**	online	https://doi.org/10.1093/database/bat0

		70
**Phyto4Health**	online	https://doi.org/10.1021/acs.jcim.2c01567
**Piel Lab DB**	unclear	https://micro.biol.ethz.ch/research/piel.html
**PubChem NPs**	online	https://doi.org/10.1186/s13321-018-0293-8
**ReSpect**	offline	https://doi.org/10.1016/j.phytochem.2012.07.007
**SANCDB**	online	https://doi.org/10.1186/s13321-015-0080-8
**Seaweed Metabolite Database (SWMD)**	online	https://www.bioinformation.net/005/007900052011.htm
**Specs Natural Products**	online	https://www.specs.net/index.php
**Spektraris NMR**	online	https://doi.org/10.1016/j.phytochem.2014.11.020
**StreptomeDB**	online	https://doi.org/10.1093/nar/gkaa868
**Super Natural II**	offline	https://doi.org/10.1093/nar/gku886
**Supernatural 3.0**	online	https://doi.org/10.1093/nar/gkac1008
**TCMDB@Taiwan (Traditional Chinese Medicine database)**	online	https://doi.org/10.1371/journal.pone.0015939
**TCMID (Traditional Chinese Medicine Integrated Database)**	offline	https://doi.org/10.1093%2Fnar%2Fgks1100
**TIPdb (database of Taiwan Indigenous plants)**	online	https://doi.org/10.1093/database/bau055
**TPPT (Toxic Plants–PhytoToxins)**	online	https://doi.org/10.1021/acs.jafc.8b01639
**UEFS (Natural Products Database of the UEFS)**	online	http://zinc12.docking.org/catalogs/uefsnp
**UNPD (Universal Natural Products Database)**	no response	https://doi.org/10.1371/journal.pone.0062839
**VietHerb**	online	https://doi.org/10.1021/acs.jcim.8b00399
**Watermelon**	online	https://doi.org/10.3389/fnut.2021.729822
**ZINC NP**	no response	https://doi.org/10.1021/acs.jcim.5b00559

Supplementary 2: Annotation Score Calculation and Value Distribution in COCONUT 2.0:

$$\text{Final Score} = \lceil 5 \times \text{round}\left((S_{\text{Literature}} \times 0.25) + (S_{\text{Organism}} \times 0.20) + (S_{\text{Collections}} \times 0.15) \right.$$

$$\left. +(S_{\text{CAS}} \times 0.15) + (S_{\text{Synonyms}} \times 0.15) + (S_{\text{Name}} \times 0.10), 2 \right) \rceil$$

Where:

- $S_{\text{Literature}}$ = 1 if citation count ≥ 1, else 0
- S_{Organism} = 1 if organism count ≥ 1, else 0
- $S_{\text{Collections}}$ = 1 if collection count ≥ 1, else 0
- S_{CAS} = 1 if CAS number exists, else 0
- S_{Synonyms} = 1 if synonyms exist and synonym count ≥ 1, else 0
- S_{Name} = 1 if name exists, else 0

Annotation score value distribution in COCONUT 2.0:

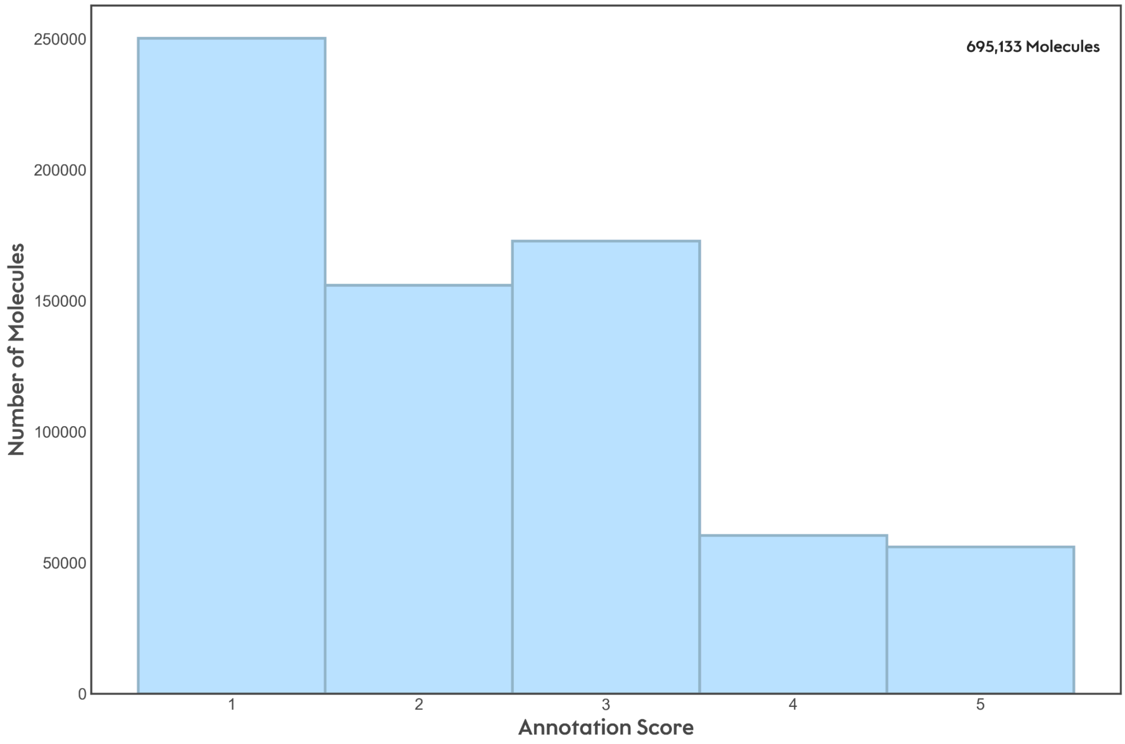

Supplementary 3: Examples of some revoked non-natural product entries in COCONUT 2.0

The database entries can still be accessed on the COCONUT web page but are flagged as "revoked" there with a detailed explanation of the reasons for the revocation. They are not included in the database exports and downloads anymore.

Compound	COCONUT_ID	Source Collections	Remarks
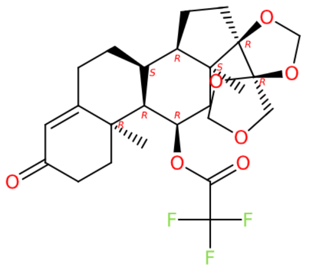<br>PubChem CID 51689994	CNP0140492.5	Super Natural II	Over 50 years have passed since the first fluorinated natural product was identified (https://doi.org/10.1016/S0022-1139(99)00201-8). During this time, only around a dozen fluorinated natural products have been isolated, with the most recent discovery occurring over a decade ago. Although there is limited knowledge about the biological fluorination mechanism, progress has been made in understanding the biosynthesis pathway of fluoroacetate and 4-fluorothreonine in Streptomyces cattleya. Prior to 1999, only six discrete fluorinated natural products had been isolated.
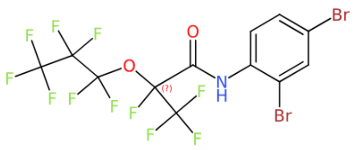<br>-{N}-(2,4-dibromophenyl)-2,3,3,3-tetrafluoro-2-(1,1,2,2,3,3,3-heptafluoropropoxy)propanamide	CNP0453484.0	Super Natural II, Super Natural III	
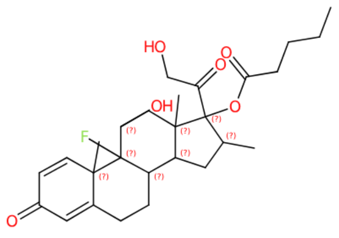<br>Betamethasone-17-valerate	CNP0335993.0	GNPS (Global Natural Products Social Molecular Networking), InterBioScreen Ltd, ZINC NP, ChEMBL NPs, NPEdia, Super Natural II	Synthetic derivative of betamethasone, a corticosteroid, which itself is a modified version of naturally occurring glucocorticoids.

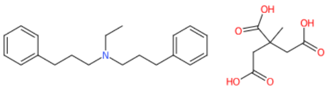<br>Alverine 2-methylpropane-tricarboxylic acid mixture	CNP0525907.0	NPEdia	Synthetic drug, chemically manufactured for its medicinal use.
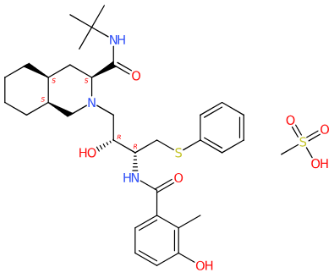<br>Nelfinavir mesylate	CNP0283515.1	GNPS (Global Natural Products Social Molecular Networking)	Fully synthetic drug designed and developed in laboratories as part of antiretroviral therapy to treat HIV.
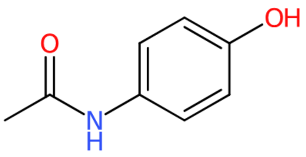<br>Acetaminophen	CNP0301653.0	ChemSpider NPs, PubChem NPs,KNApSaCK, ReSpect, StreptomeD, BitterDB, ChEBI NPs, NPASS, FooDB, NPEdia, UNPD (Universal Natural Products Database), Australian natural products,Super Natural II,EMNPD, DrugBankNP	Acetaminophen is classified as a synthetic product despite recent advances in producing it using genetically engineered microorganisms. It was first synthesised in the late 19th century. Its origin traces back to the discovery of phenacetin and acetanilide, two compounds that were explored for their fever-reducing and pain-relieving properties. The compound does not occur naturally. While microbial biosynthesis offers a more sustainable production method, this biotechnological approach does not alter its classification as a synthetic compound.

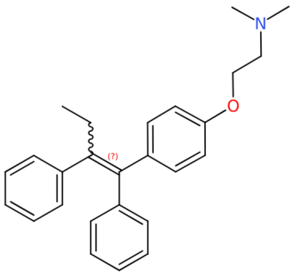<br><br>Tamoxifen	CNP0283515.2	GNPS (Global Natural Products Social Molecular Networking), ZINC NP, NPACT, StreptomeDB, NPASS, NPEdia, UNPD (Universal Natural Products Database) , Super Natural II, DrugBankNP, Super Natural III	Not a naturally occurring compound. Fully synthetic drug designed and developed in laboratories as part of antiretroviral therapy to treat HIV.
